# Warming and acidification threaten glass sponge *Aphrocallistes vastus* pumping and reef formation

**DOI:** 10.1038/s41598-020-65220-9

**Published:** 2020-05-18

**Authors:** A. Stevenson, S. K. Archer, J. A. Schultz, A. Dunham, J. B. Marliave, P. Martone, C. D. G. Harley

**Affiliations:** 1 Department of Zoology, University of British Columbia, Vancouver, British Columbia V6T 1Z4 Canada; 2 Institute for the Oceans and Fisheries, University of British Columbia, Vancouver, British Columbia Canada; 3 Marine Evolutionary Ecology, GEOMAR Helmholtz Centre for Ocean Research Kiel, Düsternbrooker Weg 20, 24105 Kiel, Germany; 4 Fisheries and Oceans Canada, Pacific Biological Station, 3190 Hammond Bay Road, Nanaimo, British Columbia V9T 6N7 Canada; 5 Louisiana Universities Marine Consortium, 8124 Highway 56, Chauvin, Louisiana 70344 USA; 6 Ocean Wise Research Institute, PO Box 3232, Vancouver, British Columbia V6B3X8 Canada; 7 Department of Botany, University of British Columbia, Vancouver, British Columbia V6T 1Z4 Canada

**Keywords:** Biodiversity, Climate-change ecology, Conservation biology, Marine biology

## Abstract

The glass sponge *Aphrocallistes vastus* contributes to the formation of large reefs unique to the Northeast Pacific Ocean. These habitats have tremendous filtration capacity that facilitates flow of carbon between trophic levels. Their sensitivity and resilience to climate change, and thus persistence in the Anthropocene, is unknown. Here we show that ocean acidification and warming, alone and in combination have significant adverse effects on pumping capacity, contribute to irreversible tissue withdrawal, and weaken skeletal strength and stiffness of *A. vastus*. Within one month sponges exposed to warming (including combined treatment) ceased pumping (50–60%) and exhibited tissue withdrawal (10–25%). Thermal and acidification stress significantly reduced skeletal stiffness, and warming weakened it, potentially curtailing reef formation. Environmental data suggests conditions causing irreversible damage are possible in the field at +0.5 °C above current conditions, indicating that ongoing climate change is a serious and immediate threat to *A. vastus*, reef dependent communities, and potentially other glass sponges.

## Introduction

Sponges have an important functional role in ecosystems worldwide and over the entire marine bathymetric gradient where they efficiently filter water, link food webs, and facilitate the flow of carbon between trophic levels^1–3^, alter the water column and its processes^4,5^, and provide biogenic habitat^6^. This is particularly true for the glass sponge *Aphrocallistes vastus* (class Hexactinellida), which – along with *Heterochone calyx* and *Farrea occa* – form large biogenic reefs that cover several square kilometres of the seafloor off the west coast of Canada^7,8^. These reefs are built through larval sponges settling atop the fused dead skeletons of previous generations, and therefore mechanical integrity of the sponge skeleton is vital to reef formation, persistence, and growth. While hexactinellids are widely distributed in the deep sea (>70 m)^9^, in British Columbia (BC), Canada, they occur as shallow as 20 m and form complex reefs, which are home to a rich community of fish and invertebrate species^6,10^. These reefs process considerable volumes of water (465–47,300 L/m^2^ per day), twice as fast as the next most intense suspension feeding community in the ocean (i.e. mussel beds), which could have strong impacts on local and regional bentho-pelagic coupling, nutrient cycling, and carbon sequestration^6,11^. These globally unique glass reefs, prior to their discovery in 1986, were thought to have been extinct for 40 million years^7^.

Although glass sponge reefs are now subject to ongoing intensive conservation efforts, responses of these and other sponge-dominated communities to ongoing environmental change remain largely unknown. Acidification could be detrimental to invertebrates, such as sponges, because of their inability to compensate for reductions in extracellular pH^12^, but responses of non-reef-building marine sponges to acidification in the lab and field are idiosyncratic and highly variable^13–18^. Also, no simple and consistent relationships have been found between temperature and pumping rate of ciliary suspension feeders^19^. Drastically different and species-specific responses to ocean acidification and warming make it difficult to predict the vulnerabilities of sponge species that have not been examined, like glass sponges. However, siliceous sponges, including hexactinellids, have survived pre-historical mass extinction events caused by ocean acidification^20,21^. Their prevalence at natural volcanic CO_2_ seeps in Papua New Guinea^22^, and persistence through rapid regional climate-induced warming along the Antarctic Peninsula^23^, suggests that siliceous sponges may do well under conditions of acidification and warming.

Here, we conducted a four-month mesocosm experiment to assess the effects of elevated temperature, CO_2_-induced acidification, and their interaction on pumping capacity, tissue withdrawal, and mechanical integrity of the skeletal structure of the reef-building glass sponge *A. vastus*. For this study, 32 juvenile specimens were randomly assigned to one of 16 aquaria (n = 2 per aquarium) set to one of four treatment combinations (4 aquaria per treatment combination): (1) control (ambient temperature = 8.6 C and pH = 7.8), (2) reduced pH (present-day temperature and projected year 2100 pH = 7.6; OA), (3) elevated temperature (projected year 2100 temperatures 10.4 C ( + 1.8 °C) and present-day pH = 7.8; OW) and (4) elevated temperature and reduced pH (projected year 2100 temperatures and pH; OAW). Fluorescent dye (Calcein) was injected near the sponge’s midsection to measure two aspects of pumping capacity: the time it took the dye to be expelled from the osculum (hereafter ‘minimum residence time’), and the vigor of the dye plume expelled from the oscula (‘pumping strength’ hereafter). Pumping strength was scored over a gradient of 0–6: ‘weak’ = a diffuse plume of dye (score 1–3), and ‘strong’ = dense plume of dye (4–6), ‘none’ = apparent pumping arrest (scored 0). ‘Apparent pumping arrest’ does not correspond to pumping arrest because a flowmeter was not used to record this, it does however infer that pumping was so weak that it was not observed with the use of a dye. Apparent pumping arrest is a common behavioral response to exposure to a stressor such as sediment^24,25^. Onset of tissue withdrawal was noted when it occurred. To assess sponge skeleton mechanical properties, we measured sponge skeleton fracture force (breaking point) and modulus (stiffness) at the end of the experiment.

## Apparent pumping arrest

**Figure 1:**
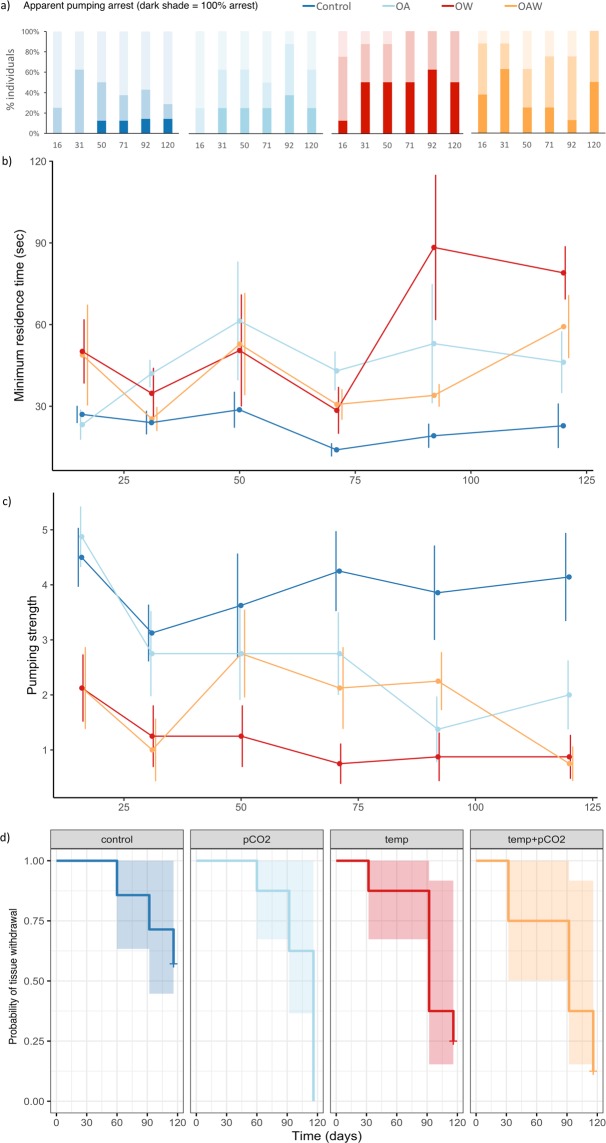
Apparent pumping arrest (**a**), minimum residence time (**b**), pumping strength (**c**), and onset of tissue withdrawal (**d**) in the reef-building glass sponge *Aphrocallistes vastus* exposed to four treatment combinations. Treatment combinations include: ambient conditions (‘Control’), CO_2_-induced acidification (‘OA’), increased seawater temperature (‘OW’), and a combination of both (‘OAW’) for four months. (**a**) Colour gradient represents total apparent pumping arrest (dark shade) to strong pumping (light shade). (**b**) ‘Minimum residence time’ refers to time (in seconds) taken to expel dye from the oscula after being injected with a fixed volume, mean values exclude individuals that were not pumping (assigned a pumping strength score of zero). (**c**) ‘Pumping strength’ is comprised of a score assigned to the volume of the plume expelled from the oscula, mean values include individuals that were not pumping (score of zero). (**d**) “Kaplan-Meier survival curve” for the probability of observing tissue withdrawal in each individual. 95% confidence limits are shown (in **d**) and error bars represent standard error (SE; in b, c) of the mean (n = 8 per treatment combination).

A greater proportion of sponges exposed to warming and/or acidification treatments ceased pumping than the control sponges over the course of the experiment (Fig. 1a), but there was no significant effect of acidification, warming, or their interaction on apparent pumping arrest over time (Table 1a). The onset of apparent pumping arrest was seen as early as two weeks in sponges exposed to warming (including OW and OAW), and the proportion of individuals not filtering remained relatively stable in the OA and OW treatments, but there were fluctuations observed in the OAW treatment combination (Fig. 1a).

**Table 1:** Results of the optimal, most parsimonious Linear and Mixed Models testing for independent and interactive effects of acidification, warming, and time (where applicable) on *Aphrocallistes vastus* performance and mechanical traits: a) apparent pumping arrest, b) minimum residence time, c) pumping strength, as well as d) the Cox proportional hazards test results for the probability of tissue withdrawal, and e) skeleton breaking force, and f) skeleton modulus (material stiffness).

Source of error	Estimate	Std. Error	z/t value	*p*-value	
**Apparent pumping arrest (Binomial GLMM)**					
Intercept	4.1732	1.5045	2.774	**0.0055**	
Acidification	−2.0661	1.6943	−1.219	0.2227	
Warming	−3.1130	1.6667	−1.868	0.0618	
Acidification × Warming	1.3895	1.9890	0.699	0.4848	
Time	−0.0212	0.0165	−1.284	0.1990	
Acidification × Time	0.0085	0.0194	0.437	0.6621	
Warming × Time	0.0076	0.0188	0.404	0.6865	
Acidification × Warming × Time	0.0101	0.0234	0.432	0.6659	
**Minimum residence time (LM, log-transformed)**					
Intercept	3.2466	0.1897	17.114	**<0.0001**	
Acidification	0.0638	0.2637	0.242	0.8093	
Warming	0.1749	0.2959	0.591	0.5555	
Acidification × Warming	−0.1127	0.4268	−0.264	0.7922	
Time	−0.0043	0.0028	−1.575	0.1178	
Acidification × Time	0.0083	0.0040	2.094	**0.0383**	
Warming × Time	0.0106	0.0043	2.448	**0.0157**	
Acidification × Warming × Time	−0.0120	0.0062	−1.930	0.0559	
**Pumping strength (Poisson GLMM)**					
Intercept	1.2967	0.2202	5.890	**<0.0001**	
Acidification	0.1080	0.3088	0.350	0.7265	
Warming	−0.6554	0.3694	−1.774	0.0760	
Acidification × Warming	0.0193	0.5029	0.038	0.9694	
Time	0.0001	0.0021	0.069	0.9451	
Acidification x Time	−0.0095	0.0034	−2.808	**0.0050**	
Warming × Time	−0.0093	0.0045	−2.062	**0.0392**	
Acidification × Warming × Time	0.0153	0.0061	2.524	**0.0116**	
**Probability of tissue withdrawal (Cox proportional hazards test)**					
**Source of error**	**Coefficient**	**Exp. Coeff**.	**Std. Err**	**z-value**	***p*-value**
Acidification	1.1961	3.3073	0.6859	1.744	0.0812
Warming	0.9815	2.6684	0.7101	1.382	0.1669
Acidification × Warming	−0.8903	0.4105	0.8803	−1.011	0.3119
**Breaking force (LM)**					
**Source of error**	**Estimate**	**Std. Error**	**t-value**	***p*-value**	
Intercept	0.1049	0.0155	6.746	**<0.0001**	
Acidification	−0.0374	0.0220	−1.703	0.1010	
Warming	−0.0479	0.0213	−2.248	**0.0336**	
Acidification × Warming	0.0451	0.0306	−1.475	0.1526	
**Modulus (stiffness) (LM)**					
Intercept	1.1803	0.1581	7.467	**<0.0001**	
Acidification	−0.5922	0.2327	−2.545	**0.0178**	
Warming	−0.4688	0.2164	−2.166	**0.0405**	
Acidification × Warming	0.5436	0.3178	1.711	0.1000	

The type of model, distribution, and transformation (where applicable) used are summarized in brackets. ‘Acidification’ = CO_2_-induced acidification. Statistically significant effects in bold; alpha = 0.05.

## Pumping capacity

Minimum residence times were similar across treatments early in the experiment, but then diverged through time in response to acidification and warming (Fig. 1b). Although minimum residence time remained constant in control tanks, it declined by 2- to 3.5- fold in sponges exposed to OA, OAW, OW treatment combinations. Individuals subjected to acidification and warming separately pumped the dye significantly slower than the control after four months (120 days) of exposure to these treatments (Table 1b). Treatment interaction dampened this negative response but not significantly.

After four months of exposure, sponges in OA, OAW, and OW tanks showed reduced pumping strength, by 2- to 5.5- fold compared to the control (Fig. 1c). Strength was significantly weaker in individuals subjected to acidification and warming relative to the control (Table 1c; Fig. 1c). Warmed sponges (OW and OAW) had depressed pumping strength as early as the first sampling point, whereas sponges in the acidification only treatment lost pumping strength more gradually (more details Supplementary Table S1). Notably, after three months, sponges exposed to elevated temperature alone showed increase in minimum residence time (slowed pumping) and decrease in pumping strength, but the pumping capacity of individuals in the OAW treatment (exposed to both acidification and warming) was relatively faster and stronger, similar to the acidified treatment. However, in the final (fourth) month pumping capacity of sponges in OAW ultimately worsened, mirroring that of individuals subjected to warming (Fig. 1). These patterns resulted in a significant Acidification x Warming x Time interaction (Table 1c).

## Tissue withdrawal

The effects of acidification and warming on tissue withdrawal were large in magnitude, but significance was not detected (Table 1d), potentially as a result of the relatively low sample size (n = 8 per treatment combination). Yet, trends are alarming and worth detailing: individuals subjected to warming (including OW and OAW treatment combinations) had earlier onset (by one month) of tissue withdrawal relative to the control and OA treatment combinations. By the end of the experiment all (100%) sponges in the OA and >75% of sponges in the warmer (OW and OAW) treatment combinations had signs of tissue withdrawal, a 35–60% increase compared to control sponges (Fig. 1d). The Cox proportional hazards regression model estimated hazard ratio (Exp. Coeff. in Table 1d) suggests a threefold increase in the probability of acidified and warmed sponges showing signs of tissue withdrawal compared to those in the control.

## Skeletal breaking force per volume and stiffness

**Figure 2:**
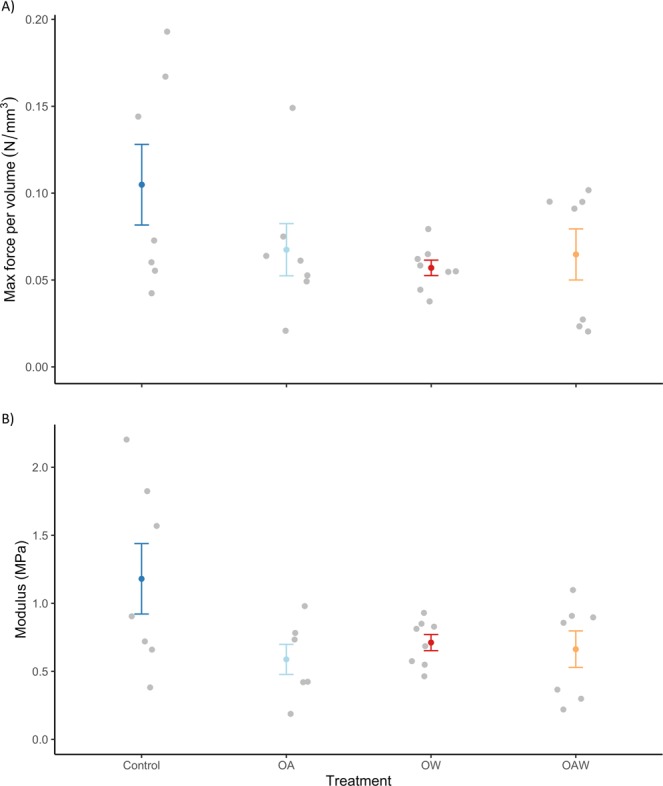
Max breaking force per volume (N/mm^3^; **a**) and mean modulus (stiffness; MPa; **b**) withstood by fresh juvenile *Aphrocallistes vastus* skeleton. Skeleton samples were tested at the end of the experiment after four months exposure to four treatment combinations: ambient conditions (‘Control’), CO_2_-induced acidification (‘OA’), increased seawater temperature (‘OW’), and a combination of both (‘OAW’). Error bars represent standard error (SE) of the mean (n = 8 sponges per treatment combination, with 5 measurements per sponge).

Experimental treatment combinations (OA, OW, OAW) reduced the force per volume required to break *A. vastus* skeleton (Fig. 2a), but only a significant effect of warming was detected (Table 1e). Both acidification and warming significantly reduced skeleton modulus (stiffness; Fig. 2b), meaning the skeleton became more elastic after four months exposure to these conditions (Table 1f). There were no significant Acidification x Warming interaction effects for these material properties.

## Warming and acidification pose an immediate threat to sponge filtration and reef formation

Our results indicate that future acidification, warming, and their combination may have substantial adverse effects on the pumping capacity, tissue withdrawal, and structural integrity of the glass sponge *A. vastus*, a species that contributes to the formation of historically and ecologically important habitats unique to the Pacific Northwest.

Most worryingly, the onset of apparent pumping arrest was quick (occurring within two weeks) for sponges exposed to elevated temperatures regardless of acidification. Subsequent degradation was observed two weeks following apparent arrest. The rigid skeleton of glass sponges, like *A. vastus*, does not permit these animals to contract like other sponges from class Demospongiae (which possess both protein and silica spicules) in response to, for example, particle obstruction^24,25^. To protect themselves from this, glass sponges typically go into temporary apparent pumping arrest whereby they cease filtering particles from surrounding water to prevent particle obstruction^24^. It is possible that treatments caused significant stress, manifested as apparent pumping arrest leading to tissue withdrawal, throughout the experiment.

Warming can influence sponge-feeding behavior by reducing choanocyte chamber density, size, and therefore filtration efficiency^26^. Few glass sponges have successfully been kept in recirculating seawater, but for those that did survive for weeks to months, unusual changes in the structure of choanocyte chambers were noted over time^27^, which may help explain tissue withdrawal observed in control sponges. To the best of our knowledge there have been no studies investigating the effects of acidification and warming on glass sponge filtration, but there are some examples among demosponges. Similar to our findings for *A. vastus, Rhopaloeides odorabile* shows drastic reductions in pumping rates and feeding efficiency in response to warming^13^. In contrast, *Dysidea avara* filtration rates remain unaffected by natural changes in temperature^28^, but in *Halichondria panicea* rates increased at higher temperatures^19^. Overall, available data suggest that ocean warming impacts on filtration capacity could be species-specific, and the effects of warming will depend on whether a particular species is already near or above its thermal optimum.

The action potential controlling filtration in glass sponge *Rhabdocalyptus dawsoni*, is known to function within a narrow temperature range (7–12 °C)^29^, but it is unclear if this is the case for other glass sponges. Below 7 °C, sponges are unable to resume filtration after arrest, and cannot undergo arrest at temperatures above 12 °C, thereby making them more susceptible to starvation and clogging from sediments^25^. The ambient and upper limit of the temperatures examined in the present study were within the physiological tolerance limits of glass sponges, but signs of distress were still observed under the climatically realistic magnitude of warming used in our experiment. It is possible that prolonged exposure (>2 weeks, as defined by our study) to warming might further restrict the physiological limits of glass sponges and could cause a decrease in biomass of *A. vastus* populations as a result of starvation (marked by apparent pumping arrest).

**Figure 3:**
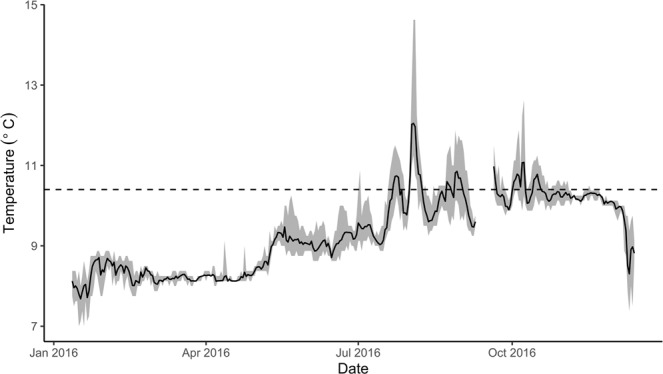
Daily average, maxima, and minima of one year (January 2016 – December 2016) iButton temperature logger data captured at the collection site, Field of a Thousand, off west Bowen Island. Dashed line represents the maximum temperature sponges experienced in the present study (10.4 °C). The recording iButton temperature logger was positioned at 23 m. (Data courtesy of Ocean Wise Research Institute).

Periods of prolonged warming have already been observed in the field, at the collection site of the present study (Fig. 3) and in other Howe Sound bioherms^30^. Warm periods, defined as temperatures reaching >10.4 C with no more than 12 hrs of cooling (temperatures < 10.4 C), lasting 6–13 days occurred six times between July and October, 2016, with five brief periods of cooling in between warm periods, which corresponded to a weak temperature anomaly (La Niña year)^31^. Results suggest that irreversible tissue withdrawal could take place in *A. vastus* after 30 days of exposure to warming (>10.4 C), which could have occurred if it were not for several brief periods of cooling observed in the summer of 2016. Warming trends pose an immediate stress to glass sponge reefs, as the addition of 0.5 °C to the 2016 pattern would result in 140 consecutive days of warming, a period longer in length and warmer than the sponges were exposed to in the present study.

Responses of glass sponges to ocean acidification have not previously been investigated; responses of species in other sponge classes are not well known and, similar to responses to warming, appear to be species-specific. Some species appear to be resistant to acidification: elevated temperatures caused significant adverse effects on abundant tropical sponge species, but acidification alone had little effect^32^. *Cliona orientalis*, a demosponge, had increased bioerosion rates under acidified conditions^14^; demosponge species were present near Mediterranean CO_2_ vent sites with pH values as low as 6.6 units^16^; no effect of acidification was found on the survival rates of *Crella incrustans*^18^; and *Mycale grandis* showed extraordinary resistance to acidification^17^. In contrast, increased mortality in response to acidification was seen in sponges *Cliona celata*^15^ and *Tethya bergquistae*^18^. Our study suggests glass sponges are less sensitive to ocean acidification than warming, at least within the range of change expected for these variables in the coming decades, but are not resilient to long-term exposure to either since both elevated temperature and acidification ultimately had detrimental outcomes for the sponges.

Importantly, the interactive effect of acidification and warming had mitigating effects on the pumping capacity of individuals exposed to warming for the first three months, mirroring the response of acidified sponges. The oscillations in pumping capacity and apparent arrest throughout the experiment suggest that the interactive effect of acidification may cause the sponges to intermittently start/stop pumping. Contrary to our work, the interaction between acidification and warming exacerbated the effect of temperature stress in heterotrophic sponge species^32^. However, acidification may mitigate these stresses in phototrophic species, reducing mortality, necrosis and bleaching of tropical sponges^32,33^. In the final month of our experiment, individuals subjected to a combination of elevated CO_2_ with warmer temperatures performed similarly to the temperature treatment suggesting that acidification may have a threshold and short-term buffering capacity. Because acidification did not dampen the presence of tissue withdrawal, it may not be able to mediate the effect of temperature and ultimate loss of this species in the long term.

It must be noted that there have been documented mass mortality of glass sponges (including *A. vastus*) in Howe Sound, where the sponges were collected for this study and several glass sponge reefs exist. These extensive glass sponge mortalities (including *A. vastus*) correlate with elevated temperatures reported during the 2009/2010 and 2015/2016 El Niño events^30,34^, and provide some indication that these sponges are sensitive to elevated temperatures. However, this period of warming was not associated with a decrease in pH^35^. Furthermore, acidification independent of warming has been documented in Howe Sound, but not associated with sponge mortalities^35^. From these field observations there can be no conclusions drawn regarding how temperature and acidification may interact in the field and how acidification may impact the sponges under natural circumstances. However, the patterns do qualitatively match the results seen in our experiment suggesting warming is the primary threat to glass sponges.

The combination of reduced skeletal stiffness (under warming and acidification) and strength (from warming) would be expected to slow or completely curtail reef formation. The fused and three-dimensional skeletal network, comprised of biosilica and chitin, held together at the joints with low concentrations of calcite, is responsible for the sponges’ rigid body that prevents disaggregation of the skeleton long after its death, allowing for reef development^36–39^. The dictyonine skeleton (fused robust scaffolding) is thought to reduce skeletal stiffness in glass sponges like *A. vastus*, providing natural flexibility to minimize stresses posed by hydrodynamic forces in shallower waters^40^. Material stiffness values (measured as Young’s modulus) from previous work on *A. vastus* are slightly higher (2.76–10.04 MPa)^40^ than those obtained in the present study (control sponges = 1.2 + 0.7 MPa). Discrepancies might be due to life stage differences as the present study was conducted on juvenile sponges (3–8 cm in height). Regardless, warmed and/or acidified sponges were half as stiff as the control sponges. Alterations to the skeleton, especially in terms of reduced stiffness (increased flexibility) as presented here, could reduce feeding efficiency, lowering the sponges’ critical water flow threshold, and potentially their distribution, restricting them to waters with higher food availability. Furthermore, under warming conditions the more brittle (measured as reduced force per volume) skeletons might collapse under the increasing weight of a growing sponge, which can reach 2–3 m in height^36^, and/or might not be able to withstand the myriad animals walking and swimming in and among the sponges^40,41^. Because we only examined material properties in living tissue, it is unclear how the dead skeleton would be altered by climate change and whether it too would succumb to the fate of the living skeleton, but it is reasonable to suspect that differences in skeletal strength apparent during life would perpetuate after death. This is critical as dead sponges are important for reef growth as larval glass sponges and other invertebrates settle and grow on the macerated skeleton^36^. The unique architecture of glass sponges vital to reef formation may be vulnerable to climate change.

## Implications for associated biodiversity and ecosystem function

Exposure to acidification and warming reduced the feeding efficiency (i.e. increased minimum residence time and decreased pumping strength) of glass sponges, suggesting that the feeding ability of juvenile *A. vastus* might be diminished (2–5.5 fold) by the end of the century as a result of climate change. Cascading effects of impaired pumping on local and regional biogeochemical processes remain unknown, but are likely to be negative. Via their remarkable filtration capacity, sponges convert large quantities of suspended particles and dissolved organic carbon (DOC) into food for other animals^1^. Through feeding, excretion, and symbiont microbial activity, sponges are known to chemically transform seawater passing through their structure^42^. The 19 documented glass sponge reefs in the Salish Sea, for example, collectively filter 1.04 × 10^11^L of water each day, representing 1% of the total water volume in the Strait of Georgia and Howe Sound combined^6^. By doing so, glass sponges bring microbial food energy from marine and terrestrial sources into local food webs by feeding on and removing up to 90% of bacteria from the water^11,43^. Reduction in this tremendous filtration capacity, as well as the reefs’ eventual physical decimation, could alter local and regional microbial loop and energy supplied to the benthic community. Examples of breakdowns in bentho-pelagic coupling exist: sponge populations in Florida Bay have historically controlled phytoplankton blooms via particle removal and pumping rates^44^. Devastation of the sponge population in the area lead to increased toxic blooms in Florida Bay. Reduced skeletal strength could act as a positive feedback loop further weakening the sponge infrastructure and making it more prone to damage from inhabitants (fish and invertebrates) moving about the reef. Habitat loss as a consequence of ocean acidification^45^ and warming^46^ has negative downstream effects on biodiversity in coral reefs, mussel beds, and some macroalgal habitats. Similarly, we anticipate biodiversity loss in these ancient glass sponge habitats as a result of climate change.

## Methods

### Collection and husbandry

Juvenile *A. vastus*, ranging in height from 3 to 8 cm, were randomly selected from ‘Field of A Thousand’ dive site on the west side of Bowen Island (49.396, −123.397) in Howe Sound, British Columbia, Canada, under collection license XR 321 2017. Sponges were placed in plastic bags with ambient seawater (collected at depth) and stored in coolers for transportation to the laboratory at the University of British Columbia. To ensure longevity, sponges were slow drip acclimatized to their tank chemistry over the course of one hour by adding 100 mL of water to the collection bag (stored in a cooler) every 10 min from the respective tank in which an individual sponge was to be housed.

Two sponges were placed in each of sixteen 250 L recirculating seawater aquaria bubbled constantly with ambient air and equipped with a multistage filtration system, including biological filter (sock filtration, protein skimmer, and bioballs) and UV sterilizer. The source seawater was obtained locally from 16 m depth in Burrard Inlet, BC, and coarse filtered by the Vancouver Aquarium. Sponges were held in total darkness with red light exposure during feeding. White light exposure was kept to a minimum, 1 hr per month or less, to measure pumping activity and observe tissue withdrawal.

Ammonia, nitrite, and nitrate (using API Marine Master Test Kit) were monitored throughout the experiment. Twenty percent water changes were performed when necessary. Water changes were also conducted at least once per month during cleaning, which was kept at a minimum to avoid stressing the animals with excessive water movement. Siliconoxide was monitored with Salifert Si Profile Test kit. To supplement silica content in the water, two drops Sponge Excel Marine High-purity Silica from Brightwell Aquatics were added twice to each tank throughout the experiment.

The sponges were fed twice daily at fixed times (every 12 hrs) to approximate their natural exposure to tidal rhythm. Each sponge tank received: in the morning, 0.5 mL Reef Nutrition Roti Feast + 0.5 mL Reef Nutrition Oyster Feast/tank mixed with 10 mL seawater, fed to the sponges using Kent Marine Sea Squirt feeder; in the evening, two drops of concentrated Sponge Power (Korallen-Zucht Sponge Power) directly added to each tank. In addition, four times weekly, 0.5 mL Fauna Marine Ultra Min S and 0.5 mL Fauna Marine Ultra Min D mixed with 10 mL seawater was added to each tank using Kent Marine Sea Squirt feeder. All food was injected near the water’s surface to prevent contact or movement near the animal.

### Experimental setup and water chemistry

The sponges were acclimated in their assigned tanks at 8–9 C for five days without food; on the sixth day, the sponges were fed and tanks were set to their experimental temperature and pH over 8 hrs. Experimental treatments were chosen based on conservative future projections (temperature + 1.8 °C and ΔpH –0.2 units based on year 2100 projections)^47^. The 16 experimental aquaria were divided equally into four treatments: (1) control (ambient temperature = 8.6 C and pH = 7.8), (2) reduced pH (present-day temperature and projected year 2100 and pH = 7.6), (3) elevated temperature (projected year 2100 temperatures 10.4 C ( + 1.8 °C) and present-day pH = 7.8) and (4) elevated temperature and reduced pH (projected year 2100 temperatures and pH). Because of a leak in the CO_2_ canister, acidification took place one week later than other treatments, but for conservative purposes its time span was treated similarly to other treatments for analyses.

Temperature was maintained using individual chillers connected to each tank. Elevated CO_2_ concentrations were achieved using mass flow controllers to bubble an appropriate mixture of compressed CO_2_ (100% CO_2_; Praxair) and ambient air (drawn from outside the building) from an air compressor. Control tanks were bubbled with ambient compressed air. Temperature was measured 5x per week using a combination of YSI (YSI Pro 30) and mercury thermometer. Seawater pH was measured 3–4x per week using Oakton pH 450 (two-point calibration with saltwater buffers AMP and TRIS, pH 6.77 and 8.09 respectively at 25 C). YSI was also used to monitor salinity.

Water samples for carbonate system parameters were collected bimonthly and stored with 10 μL Mercuric Chloride 5% (w/v) Aqueous for future analysis. Dissolved inorganic Carbon (DIC) was measured using DIC Analyzer Model AS-C3 Apollo Sci Tech, according to guidelines of the Standard Operating Procedure 2^48^. Three replicates of 0.75 mL were taken for each sample. Results were normalized to a Certified Reference Material (CRM Batch No. 154) supplied by Prof. Andrew Dickson (Scripps Institution of Oceanography). Full carbonate system parameters were conducted on the control and acidified treatments using CO2SYS^49^ (Table 2).

**Table 2:** Measured and calculated carbonate chemistry parameters for four treatment combinations: ambient conditions (‘Control’), CO_2_-induced acidification (‘OA’), increased seawater temperature (‘OW’), and a combination of both (‘OAW’).

	pH (+SD)*	Temp. (+SD)* (°C)	DIC ( + SD)* (μmol/kgSW)	TA** (μmol/kgSW)	pCO_2_** (ppm)
Control	7.83 + 0.06	8.6 + 0.3	1928 + 32	1873 + 32	919 + 104
OA	7.62 + 0.08	8.6 + 0.3	2002 + 60	1894 + 59	1525 + 234
OW	7.86 + 0.06	10.4 + 0.4	na	na	na
OAW	7.62 + 0.07	10.3 + 0.4	2002 + 53	1897 + 49	1487 + 186

Parameters of carbonate seawater chemistry (total alkalinity (TA) and pCO_2_) were calculated from measured dissolved inorganic carbon (DIC), pH, temperature, and salinity values using CO2SYS. SW – seawater; *Directly measured (n = 8 per treatment); **Calculated.

### Sponge pumping and tissue withdrawal

**Figure 4:**
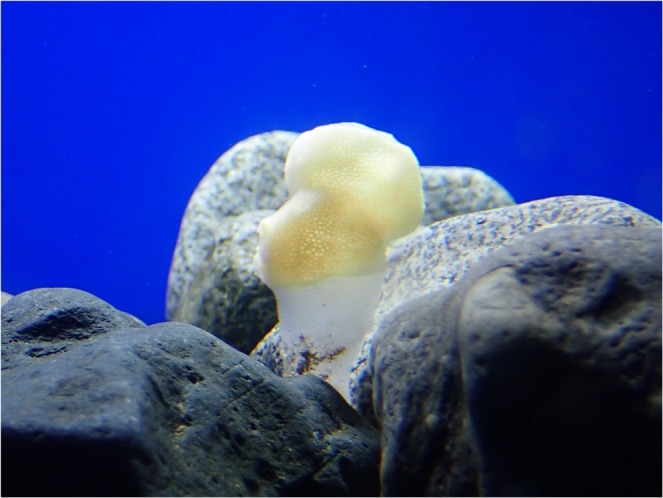
Translucent segment in lower half of *Aphrocallistes vastus* shows withdrawn tissue. Top (beige) half of sponge is comprised of living, healthy tissue.

Pumping was monitored on days 16, 31, 50, 71, 92, and 120 from the start of the experiment. Two milliliters of freshly made fluorescent Calcein dye (4 g/L; Syndel Laboratories Ltd), a fluorescent derivative of fluorescein, was injected with a pipette positioned 0.5 cm from the sponge wall and halfway down the sponge’s structure to measure pumping capacity, which was quantified by calculating the time it took the dye to be expelled from the oscula (referred to as ‘minimum residence time’ hereafter), and scoring the density of the plume expelled from the oscula (‘pumping strength’ hereafter). We calculated minimum residence time as the amount of time in between dye injection and the emergence of the dye from the sponge osculum. Here, the time it took for the dye to be expelled from a set distance was calculated not by noting the time the dye appeared above the oscula (like in the dye front method^50^) but rather when it appeared at the edge of it (distance = 0 mm from oscula) therefore a flow rate could not be calculated. We consider minimum residence time a proxy measurement for pumping rate as we were unable to calculate pumping rate directly in such small specimens. In preliminary trials we observed that sponges with similar minimum resident times differed significantly in the shape of the exhalent dye plume (see examples in Supplementary Videos S1). Consequently, we added a measurement we refer to as “pumping strength”. Video of the sponges pumping were recorded with Sony Handycam so as to precisely measure the minimum residence time. Average minimum residence time for each treatment did not include those sponges that were not pumping. Pumping strength was scored over a gradient of 0–6: ‘weak’ = a diffuse (score 1–3) and ‘strong’ = dense (4–6) plume of dye, and ‘none’ = apparent pumping arrest (scored 0). The term ‘Apparent pumping arrest’ does not correspond to pumping arrest because a flowmeter was not used to record this, it does however infer that pumping was so weak that it was not observed with the use of a dye. Scores were determined by an unbiased observer. The quantity of dye expelled, speed at which this dye was ejected, and continuous versus puffs of dye were all factors considered when scoring a plume. The presence/absence of tissue withdrawal was monitored daily until first signs of tissue withdrawal appeared. Withdrawn tissue was easily distinguished from healthy tissue by its translucent (colourless) nature whereas healthy tissue maintained its original beige or orange colour (Fig. 4). After onset, tissue withdrawal was monitored every two weeks, for the remainder of the experiment.

All sponges survived through to the end of the experiment, except one sponge in the control treatment that died in the last month of the experiment. The final time point of this sponge was excluded from the analyses because its death was deemed to be caused by a microbial infection since the sponge died suddenly (within 24 hrs) despite pumping strongly and with no signs of tissue withdrawal, and developed strings of mucus in that 24 hrs period.

### Mechanical properties

Skeleton breaking force per volume and modulus (stiffness) was tested using a standard compression method in a computer-interface tensometer (model 5500 R, Instron Corp., Canton, MA, USA). Skeleton was selected halfway down the sponge and cut into square pieces (approx. 1 cm^2^). These were placed in the cross-beam of the instrument and a maximum force of 4 N was gradually applied (load rate = 0.25 N/min; strain rate = 0.35 mm/s) to the skeleton until point of failure. Breaking force was recorded. Because thickness differed by sponge (1.7–4.6 mm), measurements of sponge skeletal thickness and cross-sectional area were used to standardize breaking force per volume. The compression surface consisted of a 3 mm diameter puncture probe. Modulus was calculated by dividing material stress with strain (i.e. the slope of the stress-strain curve produced during the compressive test). The average of 3–5 replicates was taken for analysis.

### Statistical analyses

All statistical analyses were performed in R version 3.6.0^51^ for Mac OS X. For all tests, significance was determined at p < 0.05. Data were transformed when necessary (as outlined below) to meet the assumptions of normality and equal variance. All mixed models were performed with package ‘nlme’^52^. Visual inspection of standard model validation graphs was used to verify model assumptions: residuals versus fitted values were used to verify homogeneity; a histogram or Quantile–Quantile (q–q) plot of the residuals for normality; and residuals versus each explanatory variable to check independence. Fixed and random predictor variables lacking explanatory power were eliminated via AIC model selection^53^. Optimal model structures were obtained first for random effects and next for fixed effects. To account for repeated measures (on individuals) through time, an autoregressive (AR1) correlation structure was included in the model during the selection process. In all cases, the most parsimonious model was selected (i.e., the one without interactions and/or fewer terms, least complex random structure, and removal of correlation structure where necessary).

Generalized linear mixed models (GLMM) were used to analyse apparent pumping arrest (GLMM, binomial distribution) and pumping strength (GLMM, Poisson distribution). The best model included a random intercept in which variation around that intercept depended on the sponge (nested within tank). Linear models (LM) were used on log-transformed minimum residence time, breaking force per volume, and modulus datasets. A “Kaplan-Meier survival curve” was generated using two pieces of data: status of final observation and time to event (here, first sighting of tissue withdrawal). A Cox proportional hazards regression model was used to test for the effects of acidification, warming, and their interaction on the probability of observing onset of withdrawn tissue through time, using package ‘survival’^54,55^. The Cox proportional hazard assumption was tested using Schoenfeld residuals with the function cox.zph. Sponges that did not show signs of tissue withdrawal were censored (considered to not have reached tissue withdrawal).

## Supplementary information

Supplementary Information.

Supplementary Information2.

Supplementary Information3.

Supplementary Information4.
