# Supplementary material for: Warming and acidification threaten glass sponge Aphrocallistes vastus pumping and reef formation
Source: Sci Rep. 2020 May 18;10:8176. doi: 10.1038/s41598-020-65220-9 (PMC7235243; doi:10.1038/s41598-020-65220-9)
Supplement: Supplementary file 1 — Supplementary Information. [file 41598_2020_65220_MOESM1_ESM.docx]

**Warming and acidification threaten glass sponge *Aphrocallistes vastus* pumping and reef formation**

**Stevenson A^1,2,3*^, Archer SK^4,5^, Schultz JA^6^, Dunham A^4^, Marliave JB^6^, Martone P^7^, and Harley CDG^1,2^**

^1^ Department of Zoology, University of British Columbia, Vancouver, British Columbia, V6T 1Z4, Canada

^2^ Institute for the Oceans and Fisheries, University of British Columbia, Vancouver, British Columbia, Canada

^3^ Marine Evolutionary Ecology, GEOMAR Helmholtz Center for Ocean Research Kiel, Düsternbrooker Weg 20, 24105 Kiel, Germany

^4^ Fisheries and Oceans Canada, Pacific Biological Station, 3190 Hammond Bay Road, Nanaimo, British Columbia, V9T 6N7, Canada

^5^ Louisiana Universities Marine Consortium, 8124 Highway 56, Chauvin, Louisiana, 70344 USA

^6^ Ocean Wise Research Institute, PO Box 3232, Vancouver, British Columbia, V6B3X8, Canada

^7^ Department of Botany, University of British Columbia, Vancouver, British Columbia, V6T 1Z4, Canada

* Corresponding author e-mail: stevenan@zoology.ubc.ca

**Supplementary Video S1.** Sample combinations of minimum residence time and pumping strength in glass sponge *Aphrocallistes vastus* exposed to a) CO_2_-induced acidification and increased seawater temperature (pumping strongly, minimum residence time of 10 s), b) ambient conditions (pumping strongly, minimum residence time of 21 s), c) increased seawater temperature (pumping weakly, minimum residence time of 26 s).

**Supplementary Table S1.** Pumping strength of glass sponge *Aphrocallistes vastus* exposed to four treatment combinations. Treatment combinations include: ambient conditions (‘Control’), CO_2_-induced acidification (‘OA’), increased seawater temperature (‘OW’), and a combination of both (‘OAW’) for four months. Pumping strength was scored over a gradient of 0-6: ‘weak’ = a diffuse plume of dye (score 1-3), and ‘strong’ = dense plume of dye (4-6), ‘none’ = apparent pumping arrest (cessation of pumping; scored 0). NA – data not available.

| **Sponge ID** | **Time (days)** | **Treatment** | **Pumping strength** |
| --- | --- | --- | --- |
| L11 | 16 | Control | strong |
| L11 | 31 | Control | weak |
| L11 | 50 | Control | weak |
| L11 | 71 | Control | weak |
| L11 | 92 | Control | strong |
| L11 | 120 | Control | strong |
| L12 | 16 | Control | strong |
| L12 | 31 | Control | strong |
| L12 | 50 | Control | strong |
| L12 | 71 | Control | strong |
| L12 | 92 | Control | strong |
| L12 | 120 | Control | strong |
| L7 | 16 | Control | weak |
| L7 | 31 | Control | strong |
| L7 | 50 | Control | strong |
| L7 | 71 | Control | strong |
| L7 | 92 | Control | strong |
| L7 | 120 | Control | weak |
| L9 | 16 | Control | strong |
| L9 | 31 | Control | weak |
| L9 | 50 | Control | weak |
| L9 | 71 | Control | none |
| L9 | 92 | Control | none |
| L9 | 120 | Control | none |
| R11 | 16 | Control | strong |
| R11 | 31 | Control | weak |
| R11 | 50 | Control | none |
| R11 | 71 | Control | weak |
| R11 | 92 | Control | weak |
| R11 | 120 | Control | strong |
| R12 | 16 | Control | strong |
| R12 | 31 | Control | weak |
| R12 | 50 | Control | strong |
| R12 | 71 | Control | strong |
| R12 | 92 | Control | weak |
| R12 | 120 | Control | strong |
| R7 | 16 | Control | strong |
| R7 | 31 | Control | strong |
| R7 | 50 | Control | weak |
| R7 | 71 | Control | strong |
| R7 | 92 | Control | strong |
| R7 | 120 | Control | strong |
| R9 | 16 | Control | weak |
| R9 | 31 | Control | weak |
| R9 | 50 | Control | strong |
| R9 | 71 | Control | strong |
| R9 | 92 | Control | NA |
| R9 | 120 | Control | NA |
| L17 | 16 | OA | weak |
| L17 | 31 | OA | none |
| L17 | 50 | OA | weak |
| L17 | 71 | OA | weak |
| L17 | 92 | OA | none |
| L17 | 120 | OA | none |
| L18 | 16 | OA | strong |
| L18 | 31 | OA | strong |
| L18 | 50 | OA | strong |
| L18 | 71 | OA | none |
| L18 | 92 | OA | weak |
| L18 | 120 | OA | strong |
| L19 | 16 | OA | strong |
| L19 | 31 | OA | weak |
| L19 | 50 | OA | strong |
| L19 | 71 | OA | strong |
| L19 | 92 | OA | strong |
| L19 | 120 | OA | strong |
| L20 | 16 | OA | strong |
| L20 | 31 | OA | strong |
| L20 | 50 | OA | weak |
| L20 | 71 | OA | weak |
| L20 | 92 | OA | weak |
| L20 | 120 | OA | none |
| R17 | 16 | OA | weak |
| R17 | 31 | OA | weak |
| R17 | 50 | OA | weak |
| R17 | 71 | OA | none |
| R17 | 92 | OA | none |
| R17 | 120 | OA | weak |
| R18 | 16 | OA | strong |
| R18 | 31 | OA | strong |
| R18 | 50 | OA | none |
| R18 | 71 | OA | strong |
| R18 | 92 | OA | weak |
| R18 | 120 | OA | weak |
| R19 | 16 | OA | strong |
| R19 | 31 | OA | none |
| R19 | 50 | OA | strong |
| R19 | 71 | OA | strong |
| R19 | 92 | OA | none |
| R19 | 120 | OA | weak |
| R20 | 16 | OA | strong |
| R20 | 31 | OA | weak |
| R20 | 50 | OA | none |
| R20 | 71 | OA | strong |
| R20 | 92 | OA | weak |
| R20 | 120 | OA | strong |
| L13 | 16 | OW | weak |
| L13 | 31 | OW | none |
| L13 | 50 | OW | none |
| L13 | 71 | OW | weak |
| L13 | 92 | OW | weak |
| L13 | 120 | OW | weak |
| L14 | 16 | OW | strong |
| L14 | 31 | OW | weak |
| L14 | 50 | OW | weak |
| L14 | 71 | OW | weak |
| L14 | 92 | OW | weak |
| L14 | 120 | OW | weak |
| L15 | 16 | OW | weak |
| L15 | 31 | OW | none |
| L15 | 50 | OW | none |
| L15 | 71 | OW | weak |
| L15 | 92 | OW | none |
| L15 | 120 | OW | none |
| L16 | 16 | OW | strong |
| L16 | 31 | OW | strong |
| L16 | 50 | OW | weak |
| L16 | 71 | OW | none |
| L16 | 92 | OW | none |
| L16 | 120 | OW | none |
| R13 | 16 | OW | weak |
| R13 | 31 | OW | weak |
| R13 | 50 | OW | none |
| R13 | 71 | OW | none |
| R13 | 92 | OW | weak |
| R13 | 120 | OW | weak |
| R14 | 16 | OW | weak |
| R14 | 31 | OW | weak |
| R14 | 50 | OW | strong |
| R14 | 71 | OW | weak |
| R14 | 92 | OW | none |
| R14 | 120 | OW | none |
| R15 | 16 | OW | weak |
| R15 | 31 | OW | none |
| R15 | 50 | OW | weak |
| R15 | 71 | OW | none |
| R15 | 92 | OW | none |
| R15 | 120 | OW | weak |
| R16 | 16 | OW | none |
| R16 | 31 | OW | none |
| R16 | 50 | OW | none |
| R16 | 71 | OW | none |
| R16 | 92 | OW | none |
| R16 | 120 | OW | none |
| L21 | 16 | OAW | none |
| L21 | 31 | OAW | none |
| L21 | 50 | OAW | strong |
| L21 | 71 | OAW | weak |
| L21 | 92 | OAW | strong |
| L21 | 120 | OAW | none |
| L22 | 16 | OAW | strong |
| L22 | 31 | OAW | none |
| L22 | 50 | OAW | none |
| L22 | 71 | OAW | weak |
| L22 | 92 | OAW | weak |
| L22 | 120 | OAW | none |
| L23 | 16 | OAW | weak |
| L23 | 31 | OAW | none |
| L23 | 50 | OAW | strong |
| L23 | 71 | OAW | strong |
| L23 | 92 | OAW | weak |
| L23 | 120 | OAW | weak |
| L24 | 16 | OAW | none |
| L24 | 31 | OAW | weak |
| L24 | 50 | OAW | weak |
| L24 | 71 | OAW | strong |
| L24 | 92 | OAW | weak |
| L24 | 120 | OAW | weak |
| R21 | 16 | OAW | weak |
| R21 | 31 | OAW | none |
| R21 | 50 | OAW | strong |
| R21 | 71 | OAW | none |
| R21 | 92 | OAW | weak |
| R21 | 120 | OAW | none |
| R22 | 16 | OAW | weak |
| R22 | 31 | OAW | weak |
| R22 | 50 | OAW | weak |
| R22 | 71 | OAW | weak |
| R22 | 92 | OAW | weak |
| R22 | 120 | OAW | none |
| R23 | 16 | OAW | none |
| R23 | 31 | OAW | none |
| R23 | 50 | OAW | none |
| R23 | 71 | OAW | none |
| R23 | 92 | OAW | none |
| R23 | 120 | OAW | weak |
| R24 | 16 | OAW | weak |
| R24 | 31 | OAW | strong |
| R24 | 50 | OAW | weak |
| R24 | 71 | OAW | weak |
| R24 | 92 | OAW | strong |
| R24 | 120 | OAW | weak |
